# Posterior intercostal flap: an anatomical study and best flap design

**DOI:** 10.1186/s12957-022-02711-1

**Published:** 2022-07-28

**Authors:** Ehab M. Elzawawy, Melad N. Kelada

**Affiliations:** Anatomy and Embryology Department, Faculty of Medicine, University of Alexandria, Alexandria, Egypt

**Keywords:** Posterior intercostal arteries perforators (PICAPs), Lateral intercostal arteries perforators (LICAPs), Posterior lateral perforators (PLs), Back zones, Flap design, Reconstructive surgery

## Abstract

**Background:**

Posterior intercostal arteries perforators (PICAPs) and lateral intercostal arteries perforators (LICAPs) are great vascular pedicles. Between the 4th and 11th spaces, they arise from the thoracic aorta. These are large perforators that can be the basis of many flaps. Yet, these perforators are underrated as they are poorly studied and scarcely utilized in plastic reconstructions.

**Methods:**

Twenty (ten males and ten females) adult cadaveric dissections were done on both sides to study the types, locations, and sizes of posterior intercostal perforators to help design flaps based on them in the best possible way. Perforators were assigned into one of 3 topographical zones of the back (medial, intermediate, and lateral).

**Results:**

The skin of the back was divided into 3 vertical zones: medial, intermediate, and lateral. Posterior intercostal arteries perforators (PICAPs) were found in the medial and intermediate zones. Medial zone PICAPs were large and appeared at the medial border of erector spinae (Es). Intermediate zone PICAPs appeared at the lateral border of Es and passed through latissimus dorsi (Ld) before reaching the skin. Lateral zone perforators were branches of lateral intercostal arteries and were divided into 2 types: (1) posterior branches of lateral intercostal perforators: simply named posterior lateral perforators (PLs); they were small and present in most of the spaces, and (2) anterior branches of lateral intercostal perforators (LICAPs): they were large, dominant pedicles and were found mainly in the 4th to the 7th spaces.

**Conclusion:**

PICAPs and LICAPs are constant and of enormous size and run for a great distance in the skin. They can be utilized as any type of flap.

## Introduction

The skin of the back of the trunk is supplied by the musculocutaneous branches of the posterior intercostal arteries (PICAs) and lumbar and lateral sacral arteries. PICAs are branches from the descending thoracic aorta [1]. They give PICAPs and LICAPs to the skin of the back [2].

The PICAs supply the ribs and intercostal muscles through their main stem as they run in the costal grooves. Their perforators mainly destined for the skin through direct cutaneous perforators or through musculocutaneous perforators that pass through latissimus dorsi (Ld) in the lower half of the back [2, 3].

The PICA is divided into four segments, vertebral, costal, intermuscular, and rectus, based on the neurovascular branching pattern [4]. PICAPs arise from the vertebral segment while PLs and LICAPs originate from the costal segment [5].

Hamdi et al. [4] used PICAPs to cover back defects from the lower neck to the lumbosacral area and LICAPs for breast reconstruction with great success. They stated that the flap has a great versatility and can be used to cover large defects without sacrifice of the underlying muscles.

Many studies [4, 6–9] focused on LICAPs for their importance in breast reconstruction. Few studies [4, 10] described PICAPs and PLs. We believe that these perforators are of great importance and deserve equal or even greater attention.

Bardan et al. [3] were the first to use the free intercostal perforator flaps. Prasad et al. [7] described unnamed musculocutaneous perforators that are intermediate in position between PICAPs and LICAPs and proposed to call them dorsolateral branches of PICA. Nam et al. [11] used the dorsolateral or posterolateral perforators (PLs) as a free flap to cover defects in distant sites as the face and leg.

The objective of the present work was to study the perforators of the PICAs that supply the skin of the back of the trunk regarding their location, type, length, diameter, and the possible flaps that can be based on them.

Classifications of the posterior intercostal perforators into topographical zones and comparing the results with the previous studies published concerning their detailed vascular anatomy; aiming to help and guide reconstructive and plastic surgeons in planning the possible flaps in best design that improve the use and prognosis of such flaps.

## Methods

Twenty adult cadavers’ ten males and ten females aged ranged from 25 to 65 years old with no evident trauma or operations’ scars were injected with red latex and water for better visualization of the arteries; then, the cadavers were cooled to 4 °C for 1 week before dissection to allow settling of the latex. Cadavers were dissected on both sides to identify the different vascular patterns of the PICAPs, PLs, and LICAPs from the 4th to the 11th intercostal space.

The vessels were counted, and the following measurements were taken using Vernier caliper: diameter, length, and distance from midline. Regarding location, perforators were assigned to one of 3 vertical zones (medial, intermediate, and lateral) according to their distance from the midline on the back and their relation to erector spinae (Es) muscles.

Data was collected and statistical analysis was done using Statistical Package for Social Sciences (SPSS/version 20) software. For comparison between groups, ANOVA-test was used for parametric data, followed by post hoc test and Waller-Duncan method. The level of significance was 0.05. The same small letters indicate that there was no significant difference, while different letters indicate that there was a significant difference [12].

### Ethical approval

This work was done on cadaveric specimens obtained from the dissection room of Anatomy department, Faculty of Medicine, Alexandria University.

With no violations to any rights and or ethics. All were for unknown individuals and the Alexandria faculty of medicine is the legally authorised for the cadaveric specimens and the research protocol was approved by the local ethics committee of Faculty of Medicine, Alexandria University (IRB No: 00012098- FWA No: 00018699).

## Results

**Fig. 1:**
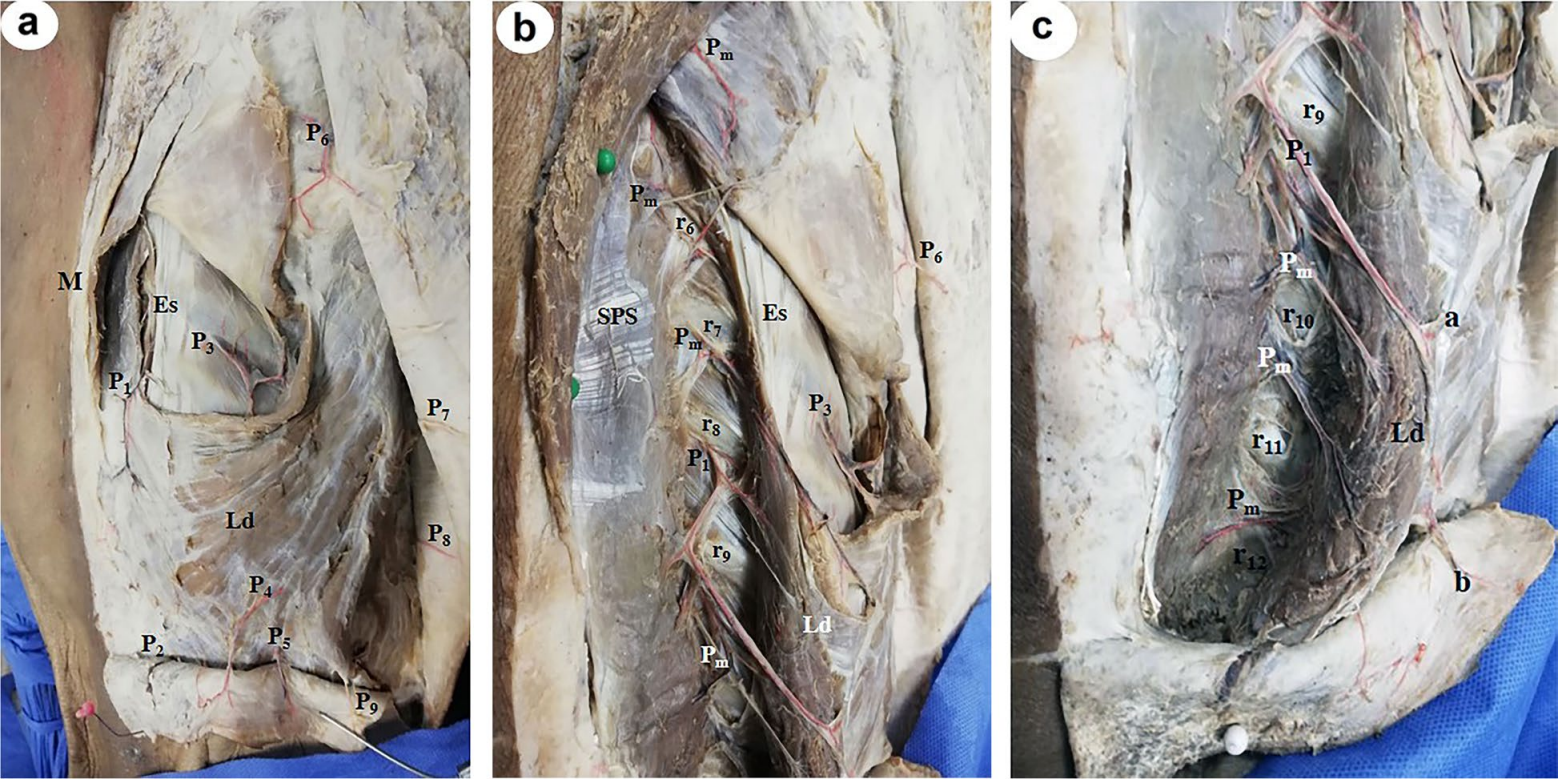
**a** A photograph of the right side of the back showing 2 perforators (P_1_ and P_2_) in the medial zone, 3 perforators in the intermediate zone (P_3_, P_4_, P_5_), and 4 perforators in the lateral zone (P_6_, P_7_, P_8_, P_9_). Medial zone perforators are accompanied by veins. Ld is latissimus dorsi muscle, Es is erector spinae muscle and M is the midline of the back. **b** A close up photograph of the upper part of the previous specimen after cutting and reflecting the vertebral origin of latissimus dorsi (Ld). P_1_ is a large perforator that comes from the 9th intercostal space and gives many branches to the surrounding muscles and skin. Several medial zone perforators (P_m_) appear above and below P_1_ and reach the skin. The erector spinae muscle (Es), serratus posterior superior muscle (SPS), the 6th to 9th ribs (r_6_ to r_9_), P_3_, and P_6_ are noted. **c** A close up photograph of the lower part of the previous specimen. P_1_ is the largest medial perforator that terminates in the skin as two terminal branches: upper one (a) and lower one (b). Several medial smaller musculocutaneous perforators (P_m_) come of the intercostal spaces below P_1_ and reach the skin. The latissimus dorsi (Ld) and the 9th to 12th ribs (r_9_ to r_12_) are noted

**Fig. 2:**
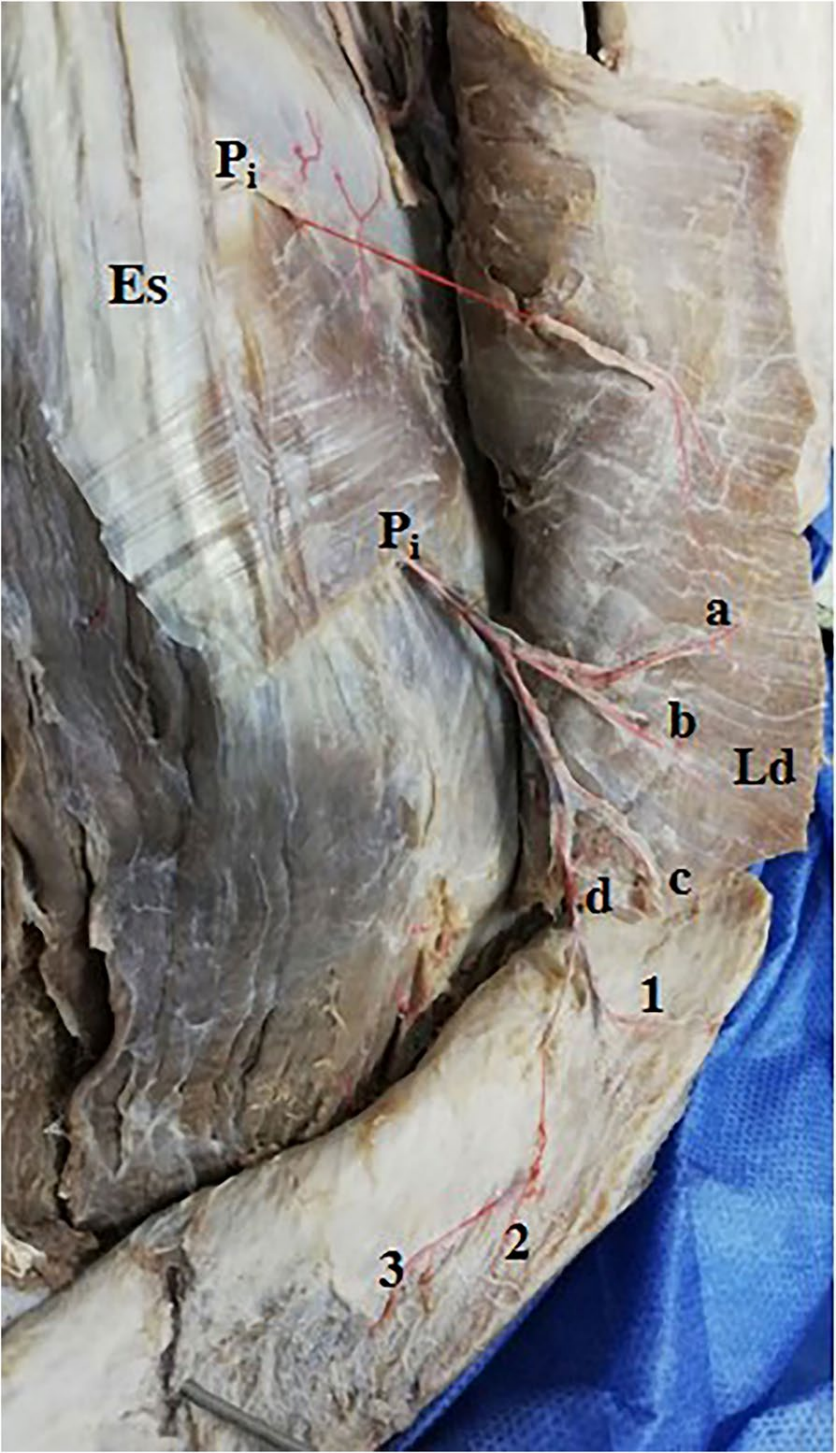
A photograph of 2 intermediate zone musculocutaneous perforators (P_i_). They come of the 6th and 7th intercostal spaces at the lateral border of erector spinae muscles (Es), pass through the back muscles, supply latissimus dorsi (Ld), and terminate in the skin (a, b, c, d). Perforator (d) further divides into 3 smaller cutaneous branches (1, 2, and 3). Note that these perforators are perpendicular to the fibers of Ld

**Fig. 3:**
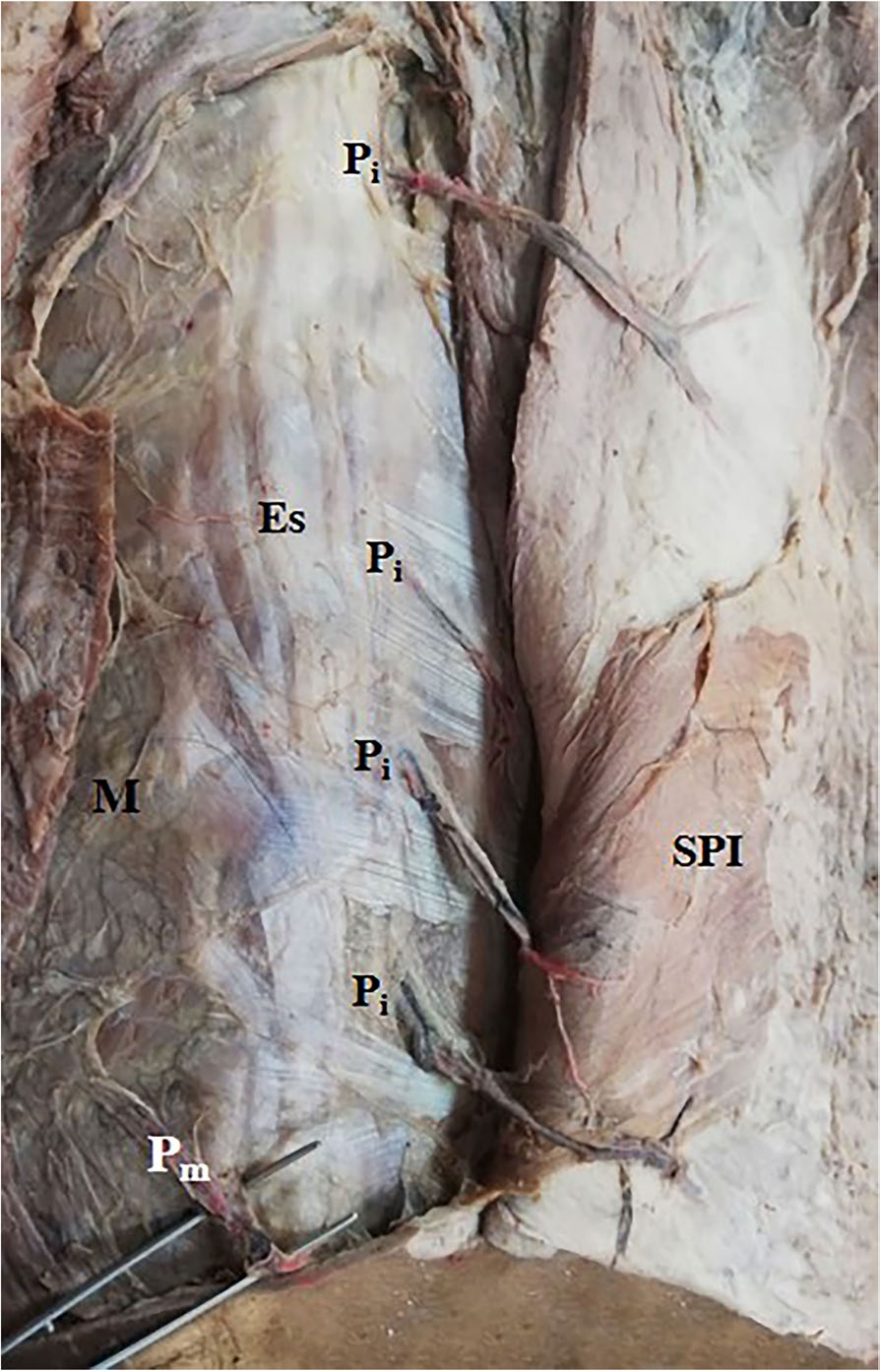
A photograph of the right side of the back showing a vertical raw of 4 intermediate zone musculocutaneous perforators (P_i_) appearing at the lateral border of erector spinae (Es). They are the 6th to 9th space perforators. They pass through the back muscles, reach the skin, and ramify into it. The 9th space medial zone perforator (P_m_) is seen at the medial border of Es. The serratus posterior inferior muscle (SPI) was reflected with the skin. M is the midline of the back

**Fig. 4:**
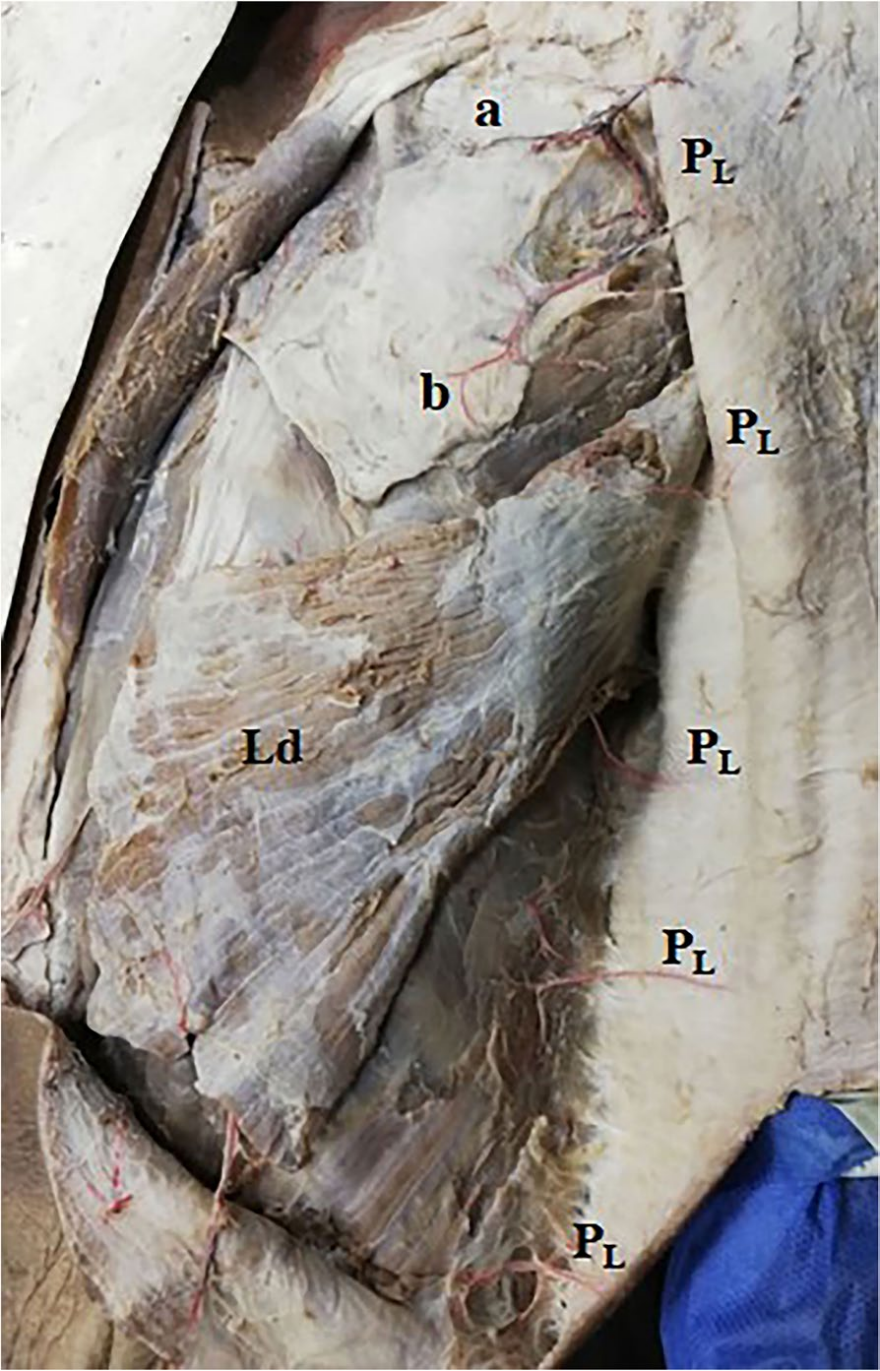
A photograph of the right side of the back showing 5 lateral zone perforators (P_L_). They come of the intercostal spaces and supply the skin as direct cutaneous perforators. The uppermost one is the largest, it comes of the 4th space and divides into an upper branch (a) and lower branch (b), and each branch gives several perforators. The latissimus dorsi (Ld) is noted

**Fig. 5:**
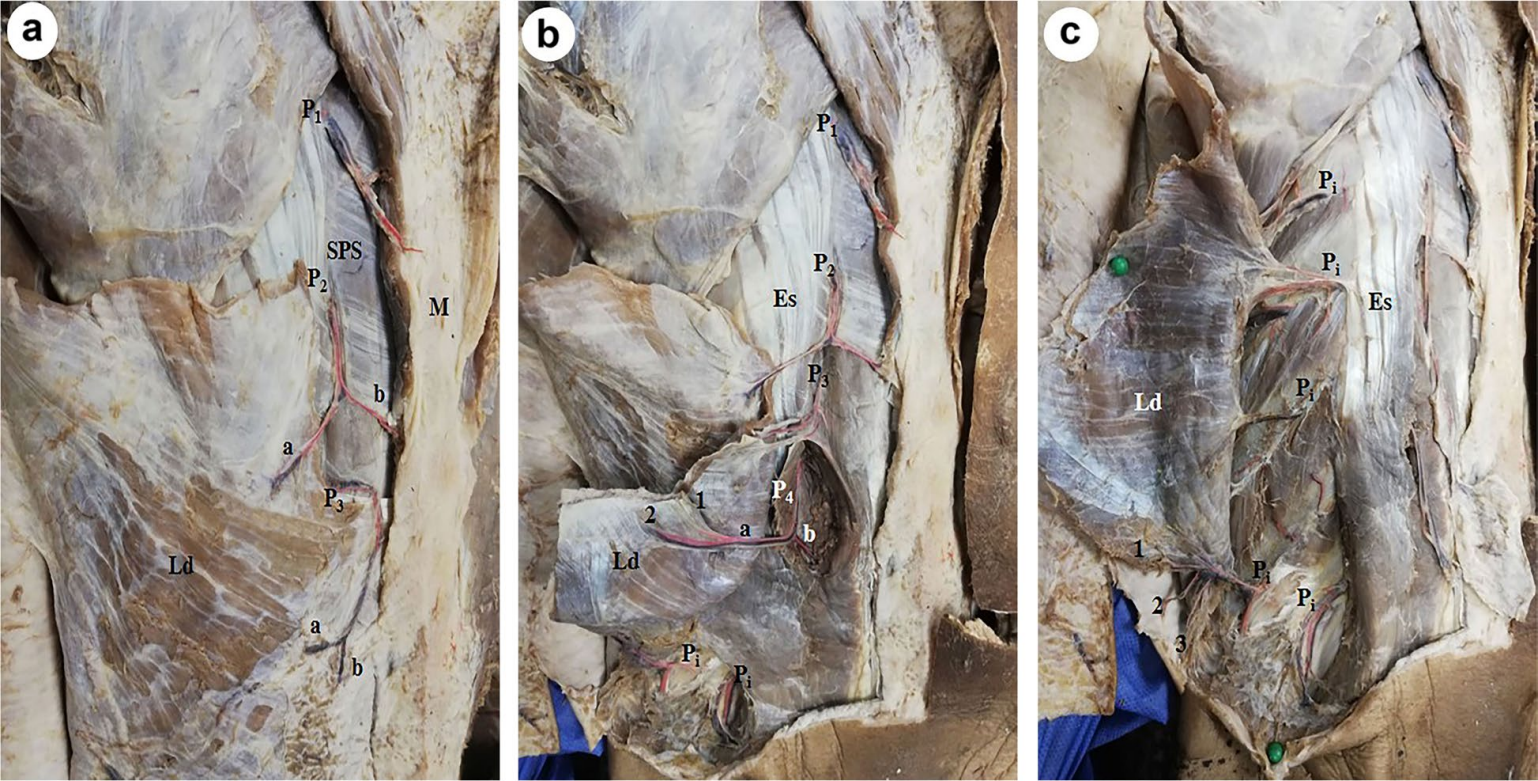
**a** A photograph of the left side of the back showing 3 medial zone perforators (P_1_, P_2_, and P3). The lower 2 perforators divide in the skin into lateral and medial branches (a, b). The latissimus dorsi (Ld), serratus posterior superior muscle (SPS), and midline of the back (M) are noted. **b** A photograph of the lower part of the previous specimen showing medial zone perforators (P_1_–P_4_) on the medial border of erector spinae muscles (Es). P_4_ is the largest and longest one; it comes of the 8th space and divides into lateral and medial branches (a, b). Branch (a) further divides into branches (1, 2) that supply latissimus dorsi (Ld) and pass to the skin. Two intermediate zone perforators (P_i_) are noted. **c** A photograph of the previous specimen after further dissection. Five intermediate perforators (P_i_) are seen at the lateral border of erector spinae muscles (Es). They supply latissimus dorsi (Ld) and reach the skin by passing through the muscles or around its lower border as perforators (1, 2, 3)

**Fig. 6:**
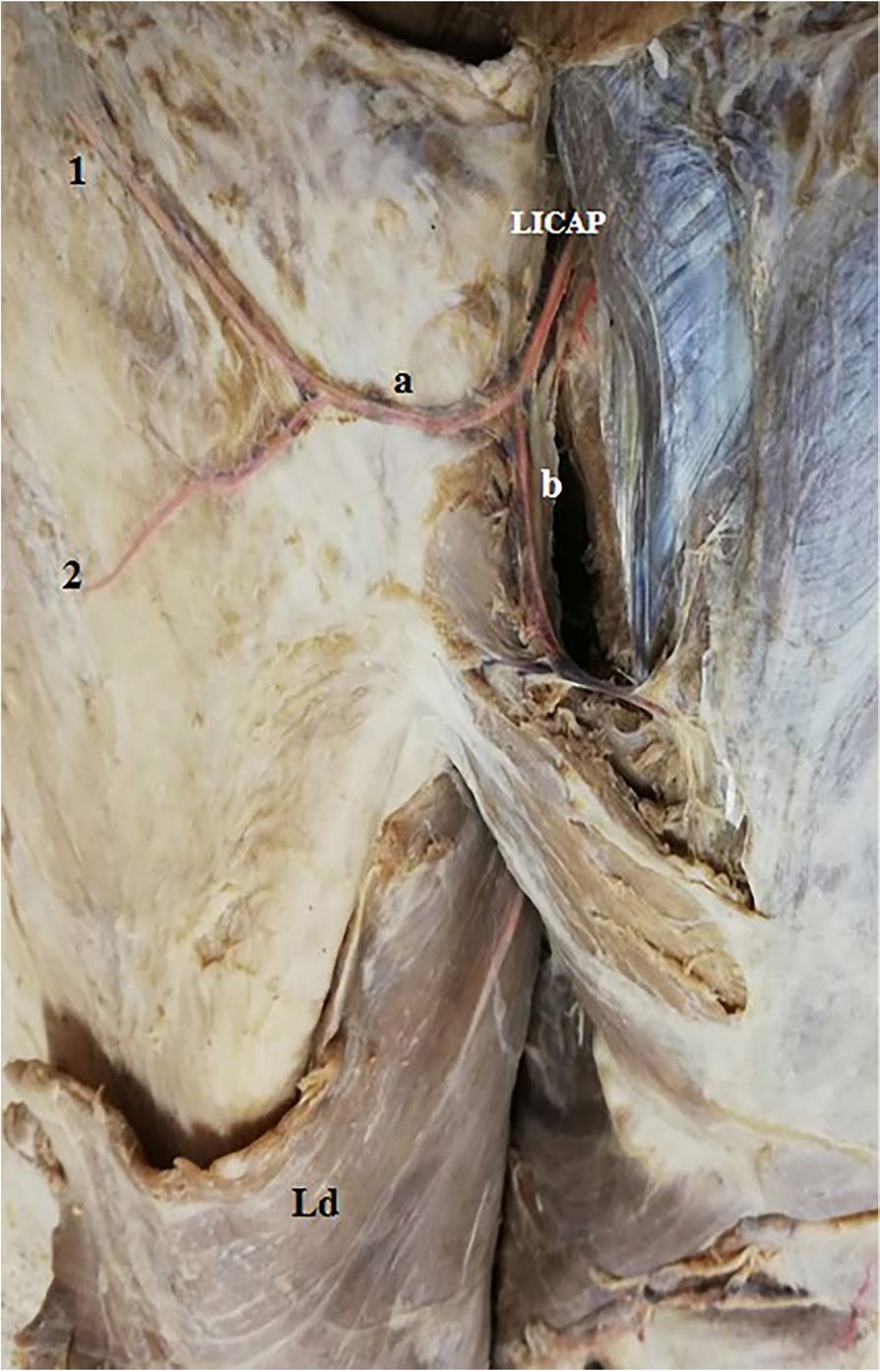
A photograph of the left side of the back. The skin of the back is dissected and reflected laterally. A large anterior branch of lateral zone perforator (LICAP) is seen coming of the 4th intercostal space. It divides into an upper branch (a) that runs a considerable distance into the skin of the upper lateral back and supplies it through 2 branches (1, 2). The lower branch (b) supplies the muscles of the back. The latissimus dorsi (Ld) is noted

**Fig. 7:**
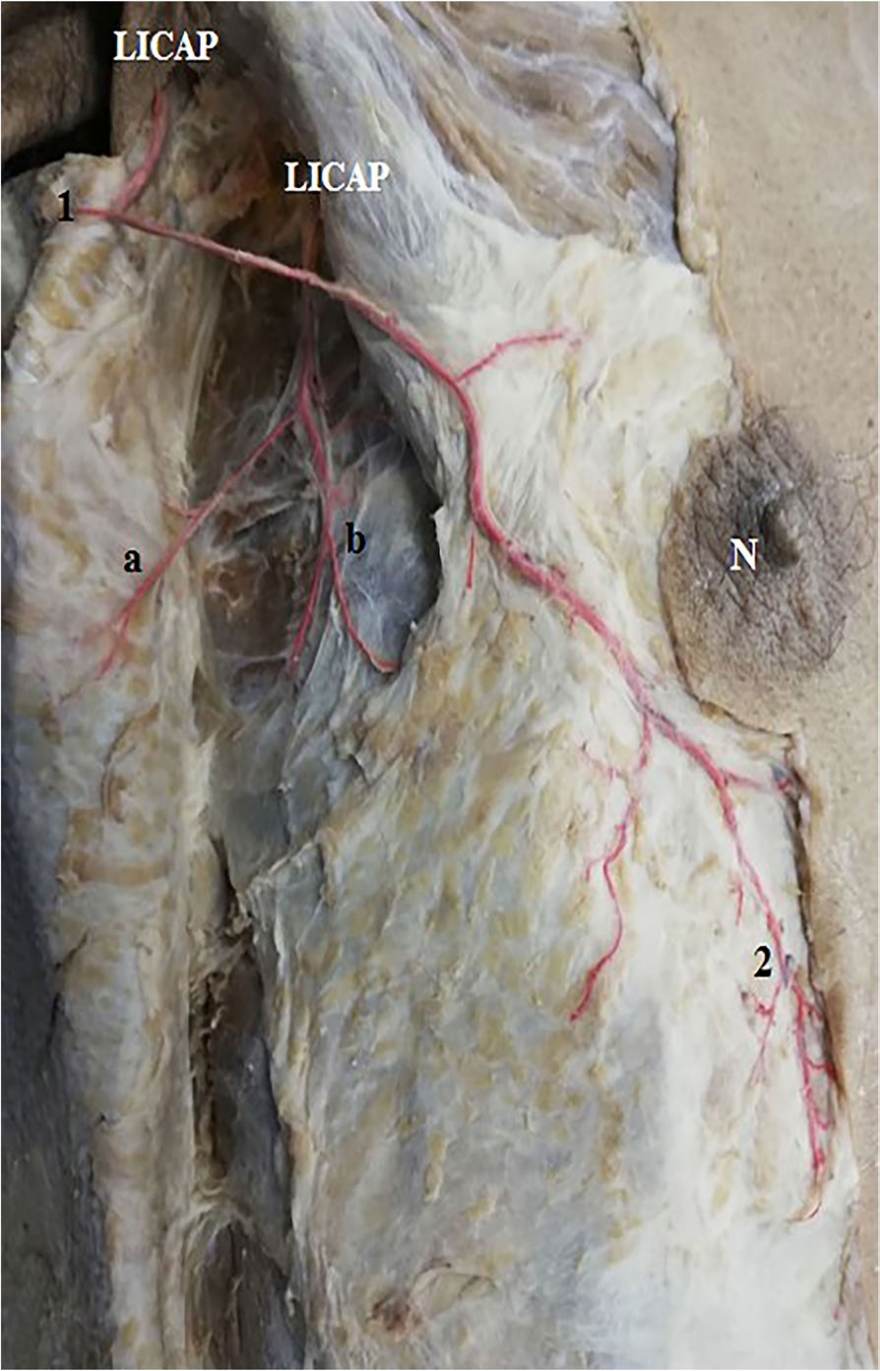
A photograph of the right lateral thoracic wall showing 2 anterior branches of lateral intercostal artery perforators (LICAP). The upper one gives branch (1) to the skin of the upper lateral back and branch (2) that reaches and supplies the submammary adipose tissue. While the lower one gives branch (a) to the skin of the back and branch (b) to muscles of lateral thoracic wall

**Fig. 8:**
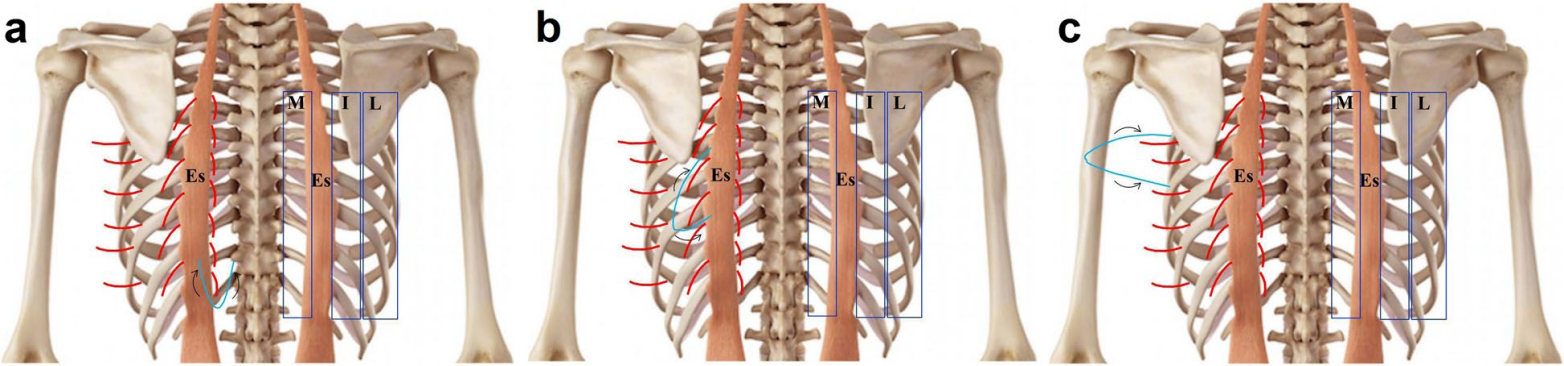
**a** A diagrammatic illustrations of the 3 perforator zones is shown on the right side: medial zone (M), intermediate zone (I), and lateral zone (L). Note the relatively bloodless line over erector spinae (Es). On the left side, note the direction of the perforators in the medial back zone and the possible flap design. **b** A diagrammatic illustrations of the 3 perforator zones is shown on the right side: medial zone (M), intermediate zone (I), and lateral zone (L). Note the relatively bloodless line over erector spinae (Es). On the left side, note the direction of the perforators in the intermediate back zone and the possible flap design. **c** A diagrammatic illustrations of the 3 perforator zones is shown on the right side: medial zone (M), intermediate zone (I), and lateral zone (L). Note the relatively bloodless line over erector spinae (Es). On the left side, note the direction of the perforators in the lateral back zone and the possible flap design

Perforators of PICA supplying the skin of the back were divided into 3 vertical zones: medial zone at the medial border of Es at a mean distance of 5±0.41cm from midline (Fig. 1a–c). Intermediate zone perforators appeared at the lateral border of Es at a mean distance of 10±0.86 cm from midline (Figs. 2, 3, 4, and 5a–c) and lateral zone perforators at a mean distance of 15±1.22cm from midline (Figs. 4, 6, 7, and 8). The perforators were found in most of the intercostal spaces from the 4th to the 11th; dominant perforators (diameter ≥ 1.5 mm) were identified in each zone.

These perforators were direct cutaneous, fasciocutaneous, and musculocutaneous. Perforators in the medial and intermediate zones were mainly musculocutaneous (Figs. 1a–c, 2, and 5b, c) while lateral zone perforators were mainly fasciocutaneous and direct cutaneous (Figs. 1a, 4, 6, and 7).

Medial zone perforators were more numerous, longer, and larger and had many branches to the skin (Figs. 1a–c, 3, and 5a, b) followed by intermediate zone perforators (Figs. 2 and 5c). Medial and intermediate zone perforators were PICAPs.

Lateral zone perforators were divided into 2 groups: (1) posterior branches of lateral intercostal perforators (we prefer to the name posterior lateral or PLs) (Figs. 1c and 4) and (2) the anterior branches of lateral intercostal perforators (LICAPs) (Figs. 6 and 7) (Tables 1 and 2).

**Table 1:** Number, total length, and diameter of perforators

Perforator	No.			Total length in cm			Diameter in mm		
	Mean±SD	Min.	Max.	Mean±SD	Min.	Max.	Mean±SD	Min.	Max.
Medial zone	7.81±1.42	6	9	10.51±1.58	7.25	12.57	1.85±0.43	1.31	2.32
Intermediate zone	6.10±1.22	4	7	8.24±1.12	6.73	9.58	1.45±0.53	1.15	1.92
Lateral zone PLs	7.50±0.62	6	8	6.13±1.21	4.01	8.12	0.81±0.35	0.64	1.23
Lateral zone LICAPs	3.42±0.83	2	5	10.42±1.26	8.55	11.66	1.92±0.64	1.40	2.45

**Table 2:** Comparison between medial, intermediate, and lateral zone perforators

Perforator	Medial zone PICAPs	Intermediate zone PICAPs	Lateral zone		***P***-value
			PLs	LICAPs	
Number	7.81±1.42^a^	6.10±1.22^b^	7.50±0.62^a^	3.42±0.83 ^c^	0.011*
Total length	10.51±1.58 ^a^	8.24±1.12^b^	6.13±1.21^c^	10.42±1.26 ^a^	0.0136*
Diameter	1.85±0.43 ^a^	1.45±0.53^b^	0.81±0.35^c^	1.92±0.64 ^a^	0.042*
Length in skin	6.88±1.50 ^a^	5.92±0.93^b^	3.81±1.03^c^	7.42±1.64 ^a^	0.033*

The same small letters indicate that there was no significant difference, while different letters indicate that there was a significant difference. ∗ means significant difference

PLs were short and small except the most upper one in the 4th space (Fig. 4) and usually passed to the skin as direct cutaneous perforators (Figs. 1a and 4). LICAPs were enormous in size; they shared in blood supply of the skin of the back and reached as far anteriorly as the submammary adipose tissue (Fig. 7). LICAPs were mainly direct cutaneous perforators (Figs. 6 and 7), (Tables 1 and 2).

The dominant medial zone PICAPs were mainly the 8th, 9th, 10th, and 11th space perforators (Figs. 1a–c and 5a, b). The dominant intermediate zone PICAPs were the 6th, 7th, 8th, and 9th space perforators (Figs. 2, 3, and 5c). The dominant lateral zone LP was the 4th space perforator. The dominant lateral zone LICAPs were the 4th, 5th, 6th and 7th space perforators (Figs. 6 and 7).

The lowermost dominant perforator was the medial zone PICAP in the 11th space (Figs. 1c and 3), while the uppermost dominant perforator was the lateral zone 4th space PL (Fig. 4).

Medial zone perforators were directed vertically downwards and reached the skin one or two spaces below their origin (Fig. 1a). They took origin from a main stem that divided into separate muscular and cutaneous branches (Fig. 1b, c).

Intermediate zone perforators were oblique to midline and oriented perpendicular to the direction of the muscle fibers of Ld and were usually present two intercostal spaces below their origin from PICA (Figs. 2 and 3). These perforators were mainly musculocutaneous as they supplied Ld and passed through it into the skin.

Lateral zone perforators were directed almost horizontally and appeared in the skin at their respective spaces (Figs. 4 and 7).

The 3 zones and the proposed flap designs are shown in Fig. 8. Relatively bloodless line is noted over Es muscle with perforators appearing at its medial and lateral borders but not through the muscle itself. It seems that the muscle is supplied by pure muscular branches that terminate into the muscle substance and do not continue into the skin unlike Ld.

## Discussion

To our knowledge, no previous study has classified posterior intercostal perforators into topographical zones. This classification is of great clinical importance and can guide the choice of a suitable flap design for reconstruction of a specific defect.

Minabe and Harii [10] found that the 4th, 5th, 6th, 10th, and 11th posterior intercostal perforators were the dominant direct cutaneous perforators. They successfully raised posterior intercostal flaps based on these vessels with a maximum flap dimension of 31 × 13 cm. They were able to locate these perforators preoperatively with great accuracy using Doppler ultrasound. These perforators were most probably medial zone perforators because they harvested them within 5cm of the spinous processes of the vertebrae.

Atik et al. [13] stated that the most common dominant perforators were the 7th and 9th space perforators as their diameters were large. They utilized PICAP flaps for closure of meningomyelocele defects. Kocak and Demir [14] describe these flaps as an ideal choice for primary closure of back defects. These vessels are equivalent to our medial zone perforators.

Prasad et al. [7] described posterior intercostal perforators in twelve cadaveric dissections. They found at least one perforator in each space and two or more perforators were in the 8th to 11th space. All perforators were found 11–12 cm from the midline and within 2 cm of the midscapular line (Table 3). They called them dorsolateral branches. These perforators correspond to our intermediate zone PICAPs.

**Table 3:** Comparison between present study and previous published studies

Study ID and year of publication	Method of perforators localization	Perforators				
		Number	Location	Length	Diameter	Zone
			cm	cm	mm	
Schmidt et al. (2019) [15]	High resolution Ultrasound	11.10±2.60	2.4±1.8	0.8±0.8	0.7±0.24	Medial
Nam et al. (2019) [11]	Cadaveric	1.65±0.67	9.79±2.04	4.82±1.07	≥2	Intermediate
Parasad et al. (2012) [7]	Cadaveric	6.4	11-12	4.6±0.4	1.85	Intermediate
Elzawawy and Kelada (2018) [16]	Cadaveric	M 7.8±1.42	M 5±0.41	10.51±1.58	1.85±0.43	Medial
		Ι 6.1±1.22	Ι 10±0.86	8.24±1.12	1.45±0.53	Intermediate
		PLs 7.5±0.62	PLs 15±1.22	6.13±1.21	0.81±0.35	Lateral
		LICAPs 3.42±0.83	LICAPs 15±1.22	10.24±1.26	1.92±0.64	Lateral

M is medial PICAPs and I is intermediate PICAPs

Nam et al. [11] used PICAP as a free flap for breast reconstruction overcoming the scar issue after thoracodorsal artery perforator (TDAP) flap. They documented that it was found between the 7th and 11th intercostal spaces. The branches were located 9.79±2.04 cm lateral to the spinous process and 9.79±2.32 cm medial to the lateral border of Ld. They only harvested perforators with a diameter of ≥2 mm. These are equivalent to our intermediate zone PICAPs (Table 3).

Jeon et al. [17] found LICAPs in 35.9% of their series, most observed in the 8th–11th intercostal spaces. Minabe et al. [18] utilized a large 10th space LICAP in adipofascial flap around split Ld pedicle for autologous breast reconstruction with great success. Kim et al. [19] used LICAPs for immediate breast reconstruction after breast conservative surgery. They stated that the most dominant perforator was the 6th, followed by the 7th space perforator. They report great cosmetic satisfaction and excellent flap survival.

From the above data, we can say that there is a considerable lack of consensus regarding location and size of perforators. There is no agreement on the location of dominant pedicles. It seems that each group of researchers has studied a certain group of perforators that can serve their flap design without looking into the whole picture.

In the present work, medial zone perforators were the largest in size and length, the most numerous, and yet the least used in reconstructive surgery. These vessels form a constant vertical raw medial to Es and are largest in the 8th to 11th spaces. They were accompanied by venae comitantes and ran a considerable distance in the skin and can be utilized in all types of flaps without muscle sacrifice as they terminate as cutaneous perforators superficial to the muscles. Medial PICAP flaps can be raised in one or more levels in the lower back perpendicular to the skin and rotated medially or laterally as needed (Fig. 8a). The only constrain in using these perforators is the difficulty in dissecting this area and the risk of compromising the blood supply of the vertebra and or the spinal cord as they share in the segmental blood supply of the cord [20].

Intermediate PICAPs appeared at the lateral border of Es. These musculocutaneous perforators were especially large in the 6th to 9th spaces. They supplied Ld and continued through the muscle into the skin. These perforators are ideal for mini latissimus dorsi flap (MLDF) and are as constant as thoracodorsal artery perforators (TDAPs) described by Elzawawy et al. [16]. Indeed, MLDF can be based on intermediate zone PICAPs sparing the rest of the muscle. Intermediate PICAP flaps can be raised at one or more levels in the middle back oblique to the skin and rotated superiorly or inferiorly as needed (Fig. 8b).

Lateral zone PLs, though small, are constantly present and pass to the skin as direct cutaneous or fasciocutaneous perforators that can be used as a local flap as it can be rotated to cover anterior or posterior defects.

LICAPs, though few, are large vessels that can be used as a free flap covering defects in far sites or as a local flap that can cover large defects especially in breast reconstruction following mastectomy with large rotational arc and guaranteed survival. LICAP flaps can be raised at one or more levels in the upper back horizontal to the skin and rotated superiorly or inferiorly as needed (Fig. 8c).

Actually, all perforators on the skin of the back can be used as local flaps or as free flaps covering defects in distant areas because of their large length and caliber.

Schmidt et al. [15] used high-resolution ultrasonography to study PICAPs. They found that the mean diameter was 0.7 ± 0.24 mm with a mean length of 0.8 ± 0.8 cm. They added that perforators were located at 2.4 ± 1.8 cm from the midline and that only 16% of all measured perforators were identified as dominant perforators (diameter ≥ 1 mm) (Table 3). Clearly this is contradicting all our results and shows how important is gross anatomical dissection to show the actual size and location of perforators. It also shows that current imaging techniques still need more enhancements to accurately detect and assess perforators.

Baghaki et al. [21] stated that although handheld Doppler examination is always useful, it is not uncommon to find perforator (s) somewhere other than the point marked with the aid of Doppler. They confirmed the consistent anatomy of PICAPs and LICAPs denoting the presence of one or two sizeable perforators always found during dissection allowing conventional, plus or propeller flap design that can cover large defects.

We can say with great certainty that PICAPs and LICAPs are present in most of the intercostal spaces from the 4th to the 11th even if they were not detected by ultrasound. Their sizes may differ between individuals, but dominant pedicles are always present. Preoperative accurate localization of these dominant pedicles with Doppler ultrasound can be done by experienced personnel and is very crucial for flap design.

Propeller PICAP flaps can be based on the dominant medial zone perforators in the 8th–11th spaces and rotated superiorly to cover upper medial back defects or rotated inferiorly to cover lower medial back defects as reconstruction following pilonidal sinus excision. The same design can be applied to intermediate zone perforators in the 6th–9th spaces as they exit Ld muscle. Propeller LICAP flaps can be based on dominant perforators in the 4th–7th and rotated anteriorly to cover lateral breast defects. These flaps have the advantage of long pedicle, and no intramuscular dissection is needed. Brunetti et al. [22] utilized propeller LICAP flaps in repair of back defects with great success emphasizing the versatility of these perforators.

Intermediate zone perforators supplying Ld can be also utilized in MLDF for early breast reconstruction following breast conservation surgery. They can be utilized in tunneled PICAP flap for delayed breast reconstruction. The flap can be tunneled through the armpit and rotated 180° to cover the defect. Brunetti et al. [23] successfully designed a tunneled PICAP flap to cover extensive cervicothoracic defects. Intermediate zone PICAP flaps can be designed in V to Y fashion to cover nearby local back defects.

Lateral zone LICAPs can be utilized in any type of flap because of their large size, easily accessible location, and redundancy of the skin at the flank. They are very useful in pedicled free flap transfer in head and neck reconstruction.

### Limitations of the current study

The main limitation of the current study was the small sample size: twenty cadavers.

## Conclusions

PICAPs and LICAPs are constant and large, give numerous branches, and cover a large cutaneous territory. They are very versatile as they can be used as pedicled flaps with large rotational arc due to their great length and consistency. They can be used in free flaps due to their large size and accompanying veins. The resultant scar is minimal if dissected along tension lines and is hidden on the back.

## Data Availability

The datasets used and/or analyzed during the current study available from the corresponding author on reasonable request.

## Abbreviations

PICAs: Posterior intercostal arteries
LICAPs: Lateral intercostal arteries perforators
PICAPs: Posterior intercostal arteries perforators
Es: Erector spinae
Ld: Latissimus dorsi
PLs: Posterior lateral perforators
SPSS: Statistical Package for Social Sciences
TDAP: Thoracodorsal artery perforator
MLDF: Mini latissimus dorsi flap
